# What Makes Consumers Purchase Fresh Eggs in Supermarkets: The Effect of Unrealistic Choice Set Matters

**DOI:** 10.3390/ani11123542

**Published:** 2021-12-13

**Authors:** Shang-Ho Yang, Widya Satya Nugraha

**Affiliations:** 1 Graduate Institute of Bio-Industry Management, National Chung Hsing University, 145 Xingda Rd., South District, Taichung 40227, Taiwan; 2 Department of Applied Economics, National Chung Hsing University, 145 Xingda Rd., South District, Taichung 40227, Taiwan; widyasatya@smail.nchu.edu.tw

**Keywords:** food choice, animal welfare, attributes, willingness-to-pay, egg

## Abstract

**Simple Summary:**

Fresh egg is a very fundamental food ingredient to consumers’ daily life, while consumers in Taiwan have faced barriers to effective decision-making for eggs they needed, including the increasing consumer concerns about food safety, traceability, and the increasing demand for animal welfare production. Consequently, Taiwanese supermarkets are eager to identify what types of fresh egg attributes would fit to their customers. Further, the effect of unrealistic options in the choice experiment design has a limited understanding. This study aims to investigate what factors affect customers’ decision-making to purchase fresh eggs in Taiwan. The findings of this research suggest that the effect of unrealistic options in the choice experiment design may mislead the results. Thus, the confirmed results showed that Taiwanese consumers are willing to pay a premium for attributes for animal welfare, traceability, farm brand, and brown color egg. Especially, the attribute of animal welfare reveals a significant effect on consumers’ decision-making. This provides an important hint to government policymakers and egg industry in Taiwan to better understand issues encompassing the diversity of attributes associated with fresh egg products offered in supermarkets.

**Abstract:**

Eggs are the crucial component of daily meals for almost everyone in Taiwan, while the multi-attributes of fresh egg products generate the challenges of marketing and promotions in supermarkets. This study analyzes the market segmentation and consumer willingness-to-pay (WTP) for fresh egg attributes (i.e., color, traceability, animal welfare, brand, and price). In particular, the effect of the unrealistic choice set is considered in this study. The data collection was distributed near markets, schools, and train stations across Taiwan from July to September in 2020. A total of 1115 valid responses were collected, and the Latent Class Model was used. Results show that fresh egg products in supermarkets reveal a strong preference for animal welfare label with the highest WTP, which is about 64.2 NT$ (≈US$ 2.29). Furthermore, traceability label, farm brand, and brown-color egg still exhibit positive WTP of about 33.4 NT$ (≈US$ 1.19), 32.6 NT$ (≈US$ 1.16), and 32.5 NT$ (≈US$ 1.16) in supermarkets, respectively. However, including the unrealistic choice set can potentially alter the final outcomes, and it provides a good example for researchers who may have the same situation. This research helps to know more about the complexity of attributes for fresh egg products in supermarkets, so marketers would be able to adopt the effective marketing strategies for fresh egg products in supermarkets.

## 1. Introduction

The egg business in Taiwan has witnessed fast expansion, with strong and rising market demand for fresh egg products in Taiwan [1]. This is shown by the growth of egg sales in Taiwan from 4% to 22% between 2015 and 2018 [2]. Further, the egg consumption per capita among Taiwanese consumers is substantial, approximately 322 eggs per person per year [3]. This implies that Taiwanese consumers consume almost one egg every day. There is a high demand for eggs because eggs are considered as one of the main staple products that can provide moderate calories (about 150 kcal per 100g) and high protein for diets [4]. Not only are eggs a good source of nutrients and protein [5,6], but also eggs can play an important agent for the human body in receiving key nutraceutical elements for the dietary [7,8,9,10]. Thus, the global guideline advocates that eggs should be consumed on a regular basis as part of a healthy diet [11]. In short, eggs are regarded as a vital element of the daily diets of all consumers [11].

Since eggs played a prominent role in consumers’ daily lives [12], the marketing strategies in supermarkets have attempted to promote many different attributes for fresh egg boxes to attract consumer attention [13,14]. Thus, it is imperative to identify consumer preference and their purchasing decision for fresh egg boxes. Previous studies [15,16,17,18,19] had focused on freshness, visual features, and prices. However, more attributes (i.e., color, brand, animal welfare, traceability, organic, and nutrition label) of fresh eggs have been discussed in other countries, but not in Taiwan [20,21,22,23].

Consumer preferences of fresh eggs may have significant differences between nations [24]. According to Li (2013) [25], Chinese consumers prefer eggs with longer shelf life and the best-before-date information. Indonesian and Indian consumers exhibit a similar preference when it comes to purchase eggs. The findings show that these consumers pay more attention on price attribute [11,26]. Further, consumers in the United States (U.S.) prefer eggs with animal welfare and organic labels [27,28]. Additionally, the majority of European consumers are more likely to buy and pay extra for eggs with animal welfare labels [29,30]. Therefore, the attributes of egg products may have a significant impact on consumers in different countries, such as China, Indonesia, the U.S., and European countries.

In general, fresh eggs in supermarkets have a series of product attributes, such as brand (providing by farm brand and private brand, e.g., Carrefour), animal welfare (giving an animal-friendly environment to enhance animal welfare for laying hens, e.g., cage-free, etc.), traceability or traceable agricultural product (providing barcodes on egg products to assist consumers in tracing and tracking the product information), and certified agricultural standard (giving a certificate that guarantees eggs are fresh, clean, food safety, etc.) [3,14,31,32]. Do more product attributes embedded on fresh eggs mean more selling? Marketers believe that product attributes would potentially satisfy consumer preferences [33]. Since more product attributes do not necessarily mean more positive influences on consumer preferences, sometimes too much information may impede decision-making and may even decrease the consumer’s desire to purchase [34,35]. Therefore, more in-depth investigation is required to determine which product attributes should be provided on fresh egg products to appropriately entice Taiwanese consumers to purchase.

Since there are a series of fresh egg attributes that will be examined and the willingness to pay (WTP) for fresh egg attributes is intended to be estimated, the choice experiments would be appropriate to implement in this study [36]. When choice experiment studies are conducted, there may exist an unrealistic situation that may not be in accordance with the actual market situations [37]. In addition to the survey design of choice experiments, the evidence of unrealistic choice sets is rare to be discussed. Since the design of choice sets for fresh egg attributes contains several unrealistic choice combinations, whether the unrealistic choice sets lead to a potential bias should be considered and discussed. Hereinafter, this study further tests the including and excluding the unrealistic choice sets to see if there is a potential bias that makes the estimator failure. Hence, this study will be able to provide more literature for the unrealistic choice sets in choice experiment research method.

## 2. Materials and Methods

A choice experiment (CE) supported by a structured questionnaire was used in this research to evaluate consumer preference, market segmentation, and willingness-to-pay (WTP) for various eggs and attribute variables in Taiwanese supermarkets. Additionally, the CE is one of the methods that can be used to reveal respondents’ preferences for different conditions, which are often restructured by researchers [38,39,40]. The target respondents in this study are the fresh egg buyers in Taiwan. The data in this study was collected through online and offline questionnaires. Finally, the data were examined using Latent Class Model (LCM), which is a statistical modeling tool that could assist researchers in calculating the prospective consumer segments and the WTP [41]. The LCM was used to ascertain which fresh egg attributes (i.e., animal welfare, brand, traceability, price, and color) should be prioritized in Taiwanese supermarkets, as well as the potential markets.

### 2.1. Questionnaire Design and Sample

In general, the methods of the CE, Conjoint Analysis (CA), and a Contingent Valuation Method (CVM) are often adopted to assess the stated preference data. The CVM method has been extensively used to estimate WTP for a specific product attribute, as well as a product that does not yet exist on the market [42]. However, the CVM is ineffective when estimating the consumer WTP with a bundle of attributes. Thus, in assessing consumer WTP for individual attribute parameters, the CE and CA are more appropriate compared to CVM [43]. However, the CE and CA still have some differences, such as the theoretical foundation. For the CE, the researchers asked respondents to select one from two or more alternatives in the questionnaire, while the researchers adopt the CA by asking the respondents to rate or rank the alternative [40]. In addition, the actual decision-making behavior is more closely to choose rather than rating or ranking. Thus, the CE method would be more appropriate to construct empirical studies for the choice paradigm. In short, the CE method is utilized to achieve the objective in this study.

Sample serves a critical role in research because it is utilized to make inferences about a population. Fresh egg buyers could be very diverse, but the majority of fresh egg consumers are those who are frequently visiting traditional markets, supermarkets, or hyper-supermarkets [14]. Those consumers may also be in charge of food grocery purchases for the family. Therefore, the sampling method should pay extra attention to selection bias [44]. This study especially focuses on fresh egg attributes on animal welfare, brand, traceability, price, and color, so the suitable respondents should be identified.

The questionnaire design in this study consists of four portions: screen questions, shopping background, the choice set questions of the CE method, and the social demographic questions. The sampling methods used in this study were offline and online. In order to fully control the selection bias and proceed with the quantitative analysis [45,46,47,48], both offline and online respondents were asked to fill out a survey link that was managed by SurveyMonkey, Inc. Thus, the survey link gave two screening questions to confirm whether respondents are targeted in this study. The first screening question is: “Are you the one in charge of grocery shopping in your family?” The second screening question is: “Have you bought any fresh raw eggs at least once in the last six months?” Respondents who are either from offline or online were able to be identified via the screening questions. The offline method was distributed in public places, such as markets, front-gate of schools, parks, and public transportation stations, and the online method was distributed through the Facebook advertisement. In order to encourage potential respondents to join the survey, a total of 150 pieces of 7–11 gift cards (valued 100 NT$ (≈US$ 3.57)/each) were provided as a lucky draw to get more responses. A total of 1555 respondents participated in our survey sampling event from July to September in 2020, while only 1115 observations are valid in this study.

### 2.2. Choice Experiments

The CE was conducted with fresh egg products in this work, as many Taiwanese consumers regularly consume approximately 322 eggs every year [3]. Hereinafter, the hypothetical discrete choice experiments in this study would determine what consumers need in the market, and their WTP for different fresh egg product attributes [49]. To mitigate the hypothetical bias, respondents in this research were encouraged to pay for the product that they would select in actual market circumstances [50]. In the CE, respondents were asked to make a choice out of different attribute alternatives [50]. In the scenario, the respondents must choose one of the options in the choice sets; however, if none of the options was of interest, they could also select a none-of-these (NOT) or no-buy option.

A no-buy option must be included in the CE model to make the decision of food purchase more realistic [51]. In addition, based on the Random Utility Theory (RUT), a respondent in the CE must choose one of many options that will maximize their utility [52]. Lancaster (1996) [53] reported, in consumer theory, the maximum utility of the product is derived from several product attributes. Thus, it is crucial to investigate the utility of each attribute in the product.

If compared to other WTP analysis methods (e.g., auctions and contingent valuation method), the advantage of the CE is that the CE format would be more similar to an actual market situation [54]. Further, this study adopts the “D-efficient” design to minimize the determinant of the covariance matrix for the parameter estimator. However, the results from the D-efficient design often produce some unrealistic combinations of attributes that contradict with the actual market situations [55]. When unrealistic choice sets exist, it is worthy to examine the differences. Thus, this study would like to investigate whether the unrealistic choices may reduce respondents’ interest in our research and cause potential biases to enlarge the estimator variance in this empirical study.

### 2.3. Attribute and Level Settings

One of the benefits of the CE model is that researchers could analyze consumer preferences for a product in the market [56]. The CE model also assigns consumer trade-offs amongst multi-attributes concepts in order to understand consumer preference [11]. There were eight choice sets produced by the R software at the time of designing the CE in this research. Before inputting the choice sets into the questionnaire, these eight choice sets were reviewed with industrial experts. There were two choice sets that indicated an unrealistic situation in light of real market circumstances. Thus, it is conceivable that if these eight choice sets can be sufficiently examined by the R software, what influence would be generated when an unrealistic choice set is included or excluded in the data analysis? As a result, this research will also examine the effect of including and excluding unrealistic CEs in the analytic model.

Additionally, this part will also discuss the design of the attributes and level settings used in this study. Next, in order to emulate a real decision-making scenario, the five attributes (i.e., brand, color, traceability, animal welfare label, and price) are often presented in supermarkets. The five attributes with different levels are shown in Table 1. The attribute of brand is defined as farm brand and private brand (i.e., Carrefour). The Carrefour private brand is the only private brand attempting to promote their fresh egg emphasizing cage-free eggs, which is similarly linked to the concept of animal welfare egg [3,57]. The color attribute has two levels, which are brown and white.

Since the Taiwanese government has enhanced food safety by promoting traceability in fresh egg products [14], the attribute of traceability is split into two levels: provided QRcode for traceability information and no QRcode for traceability information. Provided QRcode for Traceability may assist consumers to access the product information such as the farm owner’s name, location, management methods, the date of harvest, etc. Only when the chicken farm meets the government regulations will it be able to provide a QRcode for traceability. Further, the attribute of animal welfare label is defined as: provided animal welfare certified label and no animal welfare certified label. The animal welfare label is a certificate that requires an authorized third party to assess egg production meeting the regulations.

In order to evaluate consumer preferences, price is the most important factor [58]. However, the prices of fresh egg products are often different from each other in markets. In the design of the choice set, it is crucial to set the price to avoid the amount of level effect [59]. Based on market research in Taiwanese supermarkets, to avoid the amount of the level effect, the four different pricing levels were determined for one box of egg (i.e., 10 eggs): 60 NT$ (≈US$ 2.14); 95 NT$ (≈US$ 3.39); 130 NT$ (≈US$ 4.64); and 165 NT$ (≈US$ 5.89).

**Figure 1:**
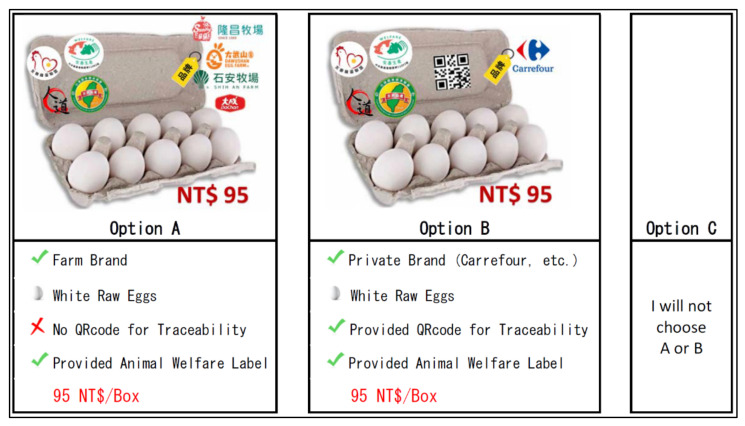
An example of a choice set—the realistic one.

The R software with Support.CEs package was used to assist the development of fractional design by selecting a profile that balances the independent effects of all egg attribute effects. Theoretically, there are 64 possible combinations that can be utilized to create the CE for this study. Following that, the total eight choice sets were finalized and created by using the “rotation.design” and “questionnaire” functions contained in the Support.CEs package. The example of a choice set is shown in Figure 1, which provides three options (option A, B, and C) and each with various attribute combinations. If respondents would not like option A or B, then they could choose option C to refuse both scenarios.

**Figure 2:**
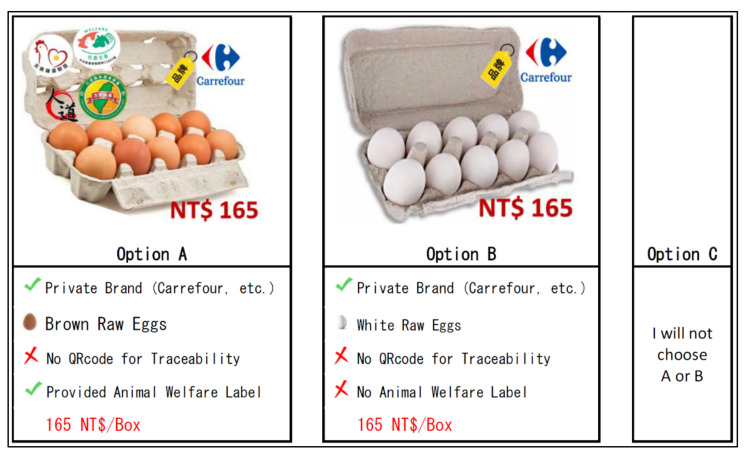
An example of an unrealistic choice question from CE.

After the set-up of choice sets, it is recommended that the choice sets should be discussed with an industrial expert to see whether the choice sets do not exist in an irrational scenario. When the choice set is excessive or unreasonable, it may influence respondents’ interest and engagement with an irrational decision as well [60,61]. Therefore, an interviewed discussion with the expert who serves in the National Animal Industry Foundation in Taiwan was implemented in June 2020. There were two choice sets identified as irrational situations, and the example is shown in Figure 2. Since a box of fresh eggs only with private brand attributes is priced at 165 NT$ (≈US$ 5.89), it was indicated by the expert that the scenario would never happen. Thus, these types of unrealistic choice sets are considered and placed as the last scenario for the CE testing.

### 2.4. Theoretical and Econometric Model Used

In general, each fresh egg product has differences in its physical characteristics, such as product packaging, size, and shell color [62]. Moreover, specific attributes contained in fresh egg products may also influence consumers while making a purchase decision [53,63]. Therefore, in order to analyze the market segmentation as well as to assess consumer preferences and the WTP for fresh egg attributes in this study, the Random Utility Theory (RUT) is adopted to describe the consumer utility of product attributes [64,65,66,67]. In addition, the RUT can play a crucial role in supporting the CE model based on the fundamental principles of economic theory [53,68].

The RUT is the model that can help researchers to capture the mobility choices of consumers. In RUT, consumers are assumed to be decision-makers, and they can often maximize their utility based on their choices or preferences [69]. Thus, in this study, it is assumed that consumers are rational and decide to optimize their utility based on the price restriction and the budget that consumers have. Further, each consumer is requested to select an alternative that was given in CE and expressed in vector notations in line with the mathematical model as:

(1) $$U_{in}=\beta_{k}\;X_{ijk}+\varepsilon_{ij}$$

where $U_{in}$ is the utility from the alternative situation “*n*” for the participant “*i*”. The homogeneous factor of the coefficient among participants can be indicated by “β” and the “$X_{ijk}$” represents the “*k*” attributes that are used in the alternative “*j*” for the “*i*” participant. The “$\varepsilon_{ij}$” presents the random residuals, which is an unknown deviation for the participant “*i*”.

### 2.5. Econometric Models

The egg-purchasing scenario in a hypothetical market was simulated using discrete choice experiments (DCE). The DCE model was selected in this study because it can assist the researchers in observing consumer preferences for fresh egg products as well as product attributes and is a well-known model among researchers [70,71]. The DCE has also been shown to be capable of predicting consumer behavior through simulating consumers’ purchasing decisions [36].

Respondents in this study were requested to choose an alternative fresh egg product that they would like to purchase or select from the scenario in the CE. Further, to express the consumer preferences, participants were forced to make a trade-off among different attributes and levels of fresh egg products in CE. Taiwanese consumers are heterogeneous in buying fresh egg products as assumed in this work [72] and different in error variation. Thus, it is crucial to consider individual preference and heterogeneity in the modeling process. Nowadays, several statistical models have been used to generate heterogeneous preferences in the DCE, such as the Latent Class Model (LCM) and the Mixed Logit Model (MLM) [73].

The LCM was used in this research to evaluate the heterogeneity of consumer preferences and market segmentation [39] for the attributes contained on fresh egg products. In order to create the CE, five attributes were selected, namely brand, color, traceability, animal welfare egg label, and price. In the LCM, participants were assumed to belong to a group with a particular probability $C_{is}$ for *s* = 1, …, *S* (where $C_{is}$ > 0, while $\sum C_{is}$ = 1; and *S* indicates the total number of groups). Hence, the probability of each class membership of the segment from the LCM will be explained in the statistical formula below:

(2) $$C_{is}=\frac{\exp\left(\alpha \lambda_{S}\right)}{\sum_{s=1}^{S}\exp\left(\alpha \lambda_{S}\right)}$$

where the vector for specific market segmentation in the LCM is represented by $\lambda_{S}$; besides this, it is also assumed that the scale factor α = 1. Thus, each respondent only has the probability of being part of a certain segment in the LCM [74].

To analyze the LCM, respondent *i*’s preference probability for alternative scenario (hypothetical market) *j* in the CE *t* can be demonstrated as below [38,39,40]:

(3) $$P_{ijt}=\overset{S}{\underset{s=1}{\sum }}C_{is}=\frac{\exp\left(\beta_{s}X_{ijt}\right)}{\sum_{j=1}^{J}\beta_{s}X_{ijt}}$$

The maximum likelihood approach is used in this study to analyze the LCM. Moreover, this model not only can estimate preferences for different consumer groups but can also provide the probability of each group or class share for each consumer group. The LCM has a function of classifying respondent *i* into a group class *s* and assumes that the stochastic error term in the membership probability function is i.i.d. (i.e., independent and identically distributed) across respondents and groups or classes.

In prior research, it has been shown that consumers have preferences that may be classified into classes or groups [39]. Therefore, the LCM model was chosen for this research since it can compute consumer preferences for each class and assume that consumer preferences differ by segments [39]. Given the membership in class *S*, the probability of membership in each latent class *S* can be calculated as the following formula:

(4) $$\pi_{c}\frac{exp\left(S_{c}+{y^{\prime }}_{c}\;Zi\right)}{\sum_{s=1}^{S}exp(S_{c}+{y^{\prime }}_{c}\;Zi)}$$

where *Zi* is the vector of variables describing respondent *i*, while *y* denotes the vector of related parameters to be estimated, and $S_{c}\;$denotes a class-specific constant. Only S−1 sets of coefficients may be recognized in the estimate for identification purposes. For the sake of identification, the vectors $y_{c}$ and $S_{c}$ are both set to zero for one arbitrary class *S*. In addition, if the covariate results are significant, the information provided in the study findings may be utilized to explain segment membership.

After the LCM results are obtained, it is essential to calculate the WTP in each market segmentation for each attribute used in the LCM. The way to analyze the WTP in the LCM is to divide each attribute’s coefficient by the coefficient of the price variable. The WTP calculation can be expressed as below:

(5) $$WTP=\frac{-2\;\beta_{\;attribute\;level}}{\beta_{\;price}}$$

## 3. Results

### 3.1. Sample Distribution

Respondents who are often in charge of grocery food purchasing for their families were targeted in this study. Particularly, respondents who had purchased fresh eggs in the past six months from supermarkets were focused on. According to the sample size calculation under the condition of the 95% confidence level [75,76], the minimum sample size is 385 observations in this study. However, although Green and Srinivasan (1978) [77] recommend that the minimum sample size is at least 100 observations to provide a credible estimate, this study, overall, collected 1115 valid respondents for this study. The sample summary of description and statistics is provided in Table 2.

Results show in Table 2 that females constituted the largest group of respondents (about 75%). Under the set-up of screening questions, this implies that most respondents in charge of grocery food purchases are female, which is identified with previous literature findings [78]. The sample’s average age is approximately 40 years old. Further, the average education year is about 15 years, which means that most respondents roughly have a bachelor’s degree education level. About 40% of respondents stated that they have a child at home. Among those occupation categories, about 14% of respondents are housewives, about 10% work in the manufacturing industry, and approximately 19% work in the service sector, while almost 6% of respondents are retired. The purchasing egg background of respondents exhibits differently in each market channel. Results reveal that respondents often go to supermarkets to purchase fresh eggs (averagely about 2.3 times in a month), while traditional markets and hypermarkets show about 1.78 and 1.11 times in a month, respectively. Thus, supermarkets are the major market channel for consumers to buy the fresh eggs they need.

### 3.2. The Determination of the Class Number in the LCM

The LCM was selected in this research to analyze the DCE choice data as well as to estimate and identify specific consumer segments for sundry attributes of eggs in supermarkets. When the LCM is utilized, the first step is to identify the number of classes. In order to decide the appropriate number of classes, the information criteria [79] are adopted in this study, such as the AIC (i.e., the Akaike Information Criteria; [80], the BIC (i.e., the Bayesian Information Criteria; [81], and the Log-Likelihood ratio index [82,83]. Swait (1994) [84] further suggested that the lowest value of AIC and BIC and the highest value of Log-likelihood would be able to identify the appropriate number of classes. 

Table 3 demonstrates the computational outcomes of AIC, BIC, and Log-Likelihood for both excluding and including unrealistic choice set models. The marginal changes in values of AIC and BIC from class 3 to 7 are very modest compared to the changes between class 2 to class 3. Further, the estimated values of the LCM for 4–7 classes are starting to deteriorate. This means that adding a class segment from 4 to 7 classes will have a potential effect, which indicates that more classes are not appropriate. In short, after trying to run LCM with several classes as well as based on the value of the Log-likelihood, AIC, and BIC, the appropriate number of classes to estimate these two LCM models is 3 classes.

### 3.3. Consumer Preferences on Fresh Egg Attributes and the Classification

In order to examine how each attribute influences consumers’ preferences, the LCM is adopted and examined in Table 4. The including unrealistic choice set model is particularly tested, and the results of AIC, BIC, and Log-Likelihood [79] reveal that the excluding unrealistic choice set model has a better goodness-of-fit than the including unrealistic choice set model. Therefore, this study will adopt the results of the excluding unrealistic choice set model as final determination, while the results of the excluding unrealistic choice set model are treated as the comparison purpose.

In this research, the LCM divides egg consumers into three categories and demonstrates that the majority of results are identical. Moreover, the researcher may examine the class share to compare classes in the LCM. As shown in Table 4, class 1 is a minority group with a market share of roughly 12.5%, class 2 is a middle group with a market share of approximately 28.3%, and class 3 is the majority group in this research with a market share of approximately 59.1%. The segmentation results indicate that there are three distinct consumer groups for fresh egg products in Taiwan supermarkets. The results in Table 4 show that the attribute of importance greatly differs among the 3 classes.

The consumers in segment 1, which represents a minority group (12.5%), show a significant disdain for almost all egg characteristics examined in this research, including farm brand and traceability. This indicates that Taiwanese consumers in this category are unconcerned with fresh egg product characteristics such as farm branding and traceability. Additionally, consumers in this segment choose private brands (carrefour) over farm brands. In addition, the estimated parameters of the color attribute have a substantial negative significance, indicating that Taiwanese consumers in this class prefer white eggs to brown eggs. The reason Taiwanese consumers favor white eggs may be related to the pricing of white eggs, which are often less expensive than brown eggs in Taiwan [85,86,87].

Respondents in class 2 (28.3% of respondents) express high preferences for attributes (brand, color, traceability, and animal welfare), but they consider the price as a monetary constraint. In this class, Taiwanese consumers prefer brown eggs as well as fresh egg products that have the farm’s brand and traceability code, as they think that the fresh egg products are safer and more reliable. Moreover, Taiwanese consumers in this category place a high premium on animal welfare labels, indicating that Taiwanese consumers prefer fresh egg products that have an animal welfare label. Thus, if we provide information about animal welfare, traceability, and farm brand on brown eggs, it may stimulate Taiwanese consumers to buy fresh egg products in Taiwanese supermarkets. Interestingly, the price variable in this class shows negative significance, indicating that although they prefer fresh egg products with animal welfare, brand, and traceability attributes, they also still prioritize price as a mandatory factor when purchasing eggs, and if the price offered is too high for them, they would not buy it as well. Therefore, it is essential to estimate how much consumers in class 2 are willing to pay for the attributes of fresh egg products.

The largest class, which is class 3 with 59.1% of the respondents, seems to prefer white eggs, farm brand label, traceability, and animal welfare label. The respondents in this class are nearly identical to class 2, in that they are more likely to have farm brand labels, traceability, and animal welfare labels. This may imply that by implementing the strategy of adding farm brand label, traceability, and animal welfare attributes into fresh egg products, it is possible that at least 87.4% percent of Taiwanese consumers will be enticed to buy fresh egg products derived from a combination of class share in classes 2 and 3.

Furthermore, the LCM membership effect is utilized to ascertain the characteristics of respondents inside each class. Table 4 indicates that the estimated parameters in the membership (socio-demographic) have an estimated value of 0, as class 3 membership is designated as the reference group in the LCM. In order to provide additional information about the effect of socio-demographic factors on the main attributes (price, brand, color, traceability, and animal welfare), this study also performs a post-estimation to predict and demonstrate the socio-demographic characteristics for each of the three classes in Table 5.

Table 5 displays the probability of class membership in each class in the LCM. Since this research focuses on the outcomes of excluding unrealistic choice sets, thus, Table 5 focuses exclusively on the findings of class membership probability in the excluding unrealistic choice sets. In terms of covariance, the estimated parameters of the female variable in Table 4 have a 10% significance with a negative coefficient, indicating that the majority of class 1 respondents are male. This is also shown in Table 5 by the findings of the LCM prediction of sociodemographic probability. It indicates that the class 1 has a higher proportion of male respondents if compared to the class 3. On the other hand, it can also be said that female respondents are relatively higher in the class 3. Regarding the age factor, respondents in the class 1 are relatively higher than those in the class 3. The education factor reveals that respondents who are in the class 3 on average have higher education than those in the class 2. The hypermarket factor does show the significant difference between the class 1 and 3.

### 3.4. The Estimation of WTP for Fresh Egg Attributes

Following Equation (5), the consumer WTP for each fresh egg attribute in the CE can be calculated and presented in Table 6. The superiority of using the LCM to analyze WTP is not only able to estimate the points for each class as well as the parameters for the entire model, but the estimation results can also be corrected with the class probability value. As explained in Table 4, the estimation outcome is focused on the excluding unrealistic choice set model, so the results of the including unrealistic choice set model is compared in Table 6.

Results of the including unrealistic choice set model in Table 6 reveal that many WTP calculations for each attribute display a significant level. However, some WTP calculations remain questions, especially for those who are in the largest consumer group. It shows that attributes of farm brand, color, and traceability have negative WTP for the largest consumer group. Since the WTP calculation is compared with the price attribute, it implies that the price attribute is the most important attribute in the largest consumer group. The middle group in Class 2 shows that consumers prefer animal welfare over price, so it shows 128.0 NT$ (≈US$ 4.57) more for animal welfare attribute. Further, other attributes of farm brand, brown-color eggs, and traceability also show that consumers in the middle group are willing to pay about 65.8 NT$ (≈US$ 2.35), 62.7 NT$ (≈US$ 2.24), and 51.7 NT$ (≈US$ 1.85) for attributes of traceability, brown-color eggs, and farm brands, respectively. However, consumers in the smallest consumer group of Class 1 would like to pay about 22.2 NT$ (≈US$ 0.79) for the traceability attribute. Although the results of the including unrealistic choice set model may represent any potential situation, the final WTP results would not adopt it from this model in order to not over-explain the outcomes in this study.

Furthermore, the excluding unrealistic choice set model results indicate that class 2, which is the middle group (28.3% of respondents), is the only segment for which the estimated parameters are significant in terms of mean willingness-to-pay (MWTP). According to Table 6, Taiwanese consumers want to pay about 32.6 NTD (≈US$ 1.16) more for farm brand labels than private brand labels such as Carrefour. The result indicates that farm brand is becoming more well-received in Taiwan, as Taiwanese consumers are starting to pay more attention and support local farmers in Taiwan. Additionally, Taiwanese consumers are willing to pay an additional 32.5 NTD (≈US$ 1.16) for brown eggs to white eggs. Hereinafter, Taiwanese consumers are willing to pay an over 33.4 NTD (≈US$ 1.19) premium for fresh egg products with traceability labels. On average, the highest WTP of the egg attributes is constituted by animal welfare at 64.2 NTD (≈US$ 2.29).

Although the majority group did not show a significant positive WTP in Table 6, they showed a positive response to the farm brand, traceability, and animal welfare variables in Table 4. The estimated parameters for the farm brand, traceability, and animal welfare attributes in class 3 did not show the positive correlation with MWTP, due to Taiwanese consumers’ lack of knowledge about those attributes. Hence, government assistance is needed to promote the attributes of farm brand, traceability, and animal welfare; thus, Taiwanese consumers will be more familiar with those attributes. Furthermore, as the results indicate that brown eggs with farm brand, traceability, and animal welfare attributes have a positive response from Taiwanese consumers, this can be further confirmed and provide a hint to the Taiwanese government and egg industry that by providing farm brand, traceability, and animal welfare attributes on fresh egg products, it may influence Taiwanese consumers to purchase fresh eggs.

## 4. Discussion

The present research used DCEs to ascertain which attributes may affect Taiwanese consumers’ decisions to buy fresh eggs in Taiwanese supermarkets, and five attributes of fresh eggs were considered: price, brand, color, traceability, and animal welfare. This researcher conducted an unrealistic choice experiment on CE to evaluate the impact of the unrealistic choice set on the estimated findings. Thus, this research will serve as a reference for including or excluding the unrealistic choice experiment for the CE design. Furthermore, this study also contributes significantly to the Taiwan government, egg business, and policymakers’ in terms of heterogeneous consumer preferences for egg attributes in Taiwanese supermarkets. Using the LCA method, this research found three unique groups of Taiwanese consumers, each with its own distinct set of preferences and WTP for fresh egg attributes, as well as distinct sociodemographic and behavioral traits.

As shown by the AIC, BIC, and Log-likelihood values in Table 3, the data set without unrealistic options is more suitable than the data set with unrealistic choices as it has a greater Log-likelihood and lower AIC and BIC values [79]. Moreover, Table 3 also shows that 3 classes in the LCM gave the best fit since the marginal changes in AIC and BIC values between classes 3–7 are extremely small in comparison to the changes between classes 2 and 3. Additionally, the model’s estimated value for classes 4–7 have begun to degrade, resulting in an unstable AIC and BIC value. To summarize, the optimal number of classes for estimating LCM models is three.

Table 4 presented and contrasted the findings of LCM analysis on two distinct data types in order to determine the most appropriate data set for assisting the researchers in accurately analyzing and interpreting this study’s results. Further, this study will determine whether or not an unrealistic choice set has an effect on the LCM estimate outcome. The goodness of fit value generated by the LCM analysis may be evaluated [79] to determine which data sets are more fitted and perform better with the model in this research. The AIC, BIC, and Log-likelihood values in Table 4 suggest that excluding the unrealistic choice set model is more appropriate than excluding the unrealistic choice set model.

The results of attributes in Table 4 show that most outcomes are similar to each other. The class shares of the excluding unrealistic choice set model reveal that the class-3 is the biggest consumer group with about 59% market share, while the middle and small groups of class share exhibit about 28% and 12%, respectively. It means that there roughly can present three types of consumer groups for fresh egg products in supermarkets. The attribute preferences of the largest consumer group show that they prefer farm brand, white-color eggs, traceability, and animal welfare. However, the middle-group consumers prefer lower prices, farm brand, brown-color eggs, traceability, and animal welfare; and the small-group consumers are particularly like the private brand of supermarkets, white-color eggs, and no traceability label. This implies that there still is a small consumer group that does not really prefer any attribute.

In addition, the results of the including unrealistic choice set model show that the largest consumer group with about 45% market share, while the middle and small groups of class share reveal about 40% and 15%, respectively. The composition of market shares in the including unrealistic choice set model is similar to the excluding unrealistic choice set model. If the traceability is compared in the small consumer group, then it received a different result. It implies that the smallest consumer group reveals a positive preference for traceability. Further, the results of attributes in the largest consumer group show that consumers prefer higher prices, farm brand, white-color eggs, and traceability. This is a bit of a contradictory outcome in price, since it implies that consumers will tend to buy more eggs if the price is higher. This outcome may reflect the cause of unrealistic choice sets. Moreover, if the unrealistic choice sets exist, it may lead to an expected outcome like this. Therefore, it is an important finding in this study that provides a good example if future studies adopt the CE method. However, the results of including unrealistic choice sets are not adopted as the final outcome in this study.

Following Equation (4), Table 4 also shows how each social demographic variable and shopping background contribute to each class share. The largest consumer group of the class-3 is compared. Results of the excluding unrealistic choice set model show that higher age male consumers who usually purchase fresh eggs from non-hypermarkets are more likely to be in the smallest consumer group (i.e., the class-1). It also means that these consumers prefer attributes of private band, white-color eggs, and no-traceability when comparing to those who are in the largest consumer group. Although previous studies [85,86,87] mentioned that the preference for white eggs is because it is cheaper than brown eggs, consumer preferences of white eggs in the class-1 do not link to the price attribute. It can be confirmed that some consumers may still prefer white eggs. The reason Taiwanese consumers prefer white eggs may correlate with the eggs price as usually, the price of white eggs in Taiwan tends to be cheaper than brown eggs. Consumers with lower education would prefer attributes of a lower price, farm brand, brown-color eggs, traceability, and animal welfare if compared to a reference group. Thus, it can be identified that younger female consumers with higher education who usually shopped at hypermarkets tend to be in the largest consumer group. This also corresponds to previous findings [14,31], that there is a potential market trend on-farm brand, traceability, and animal welfare for the majority of consumers.

The final WTP results will not contain an unrealistic option set model to avoid over-explaining the WTP findings of this study, even though the results may reflect every possible scenario. Thus, in order to account for the WTP findings in this study, the researcher concentrates only on excluding the unrealistic choice set model.

The WTP results of the excluding unrealistic choice set model reveal that only the middle consumer group of class-2 shows a significant level with a positive sign. This implies that consumers in the middle group (28.3% of market share) are more likely to pay more for these attributes than the price attribute; in other words, consumers are willing to pay more for the attributes of farm brand, brown-color eggs, traceability, and animal welfare. Particularly, consumers in the middle group would like to pay about 32.6 NT$ (≈US$ 1.16) more for farm brand labels if compared to the private brand label (i.e., Carrefour). This indicates that fresh eggs with farm brands are receiving more attention than the private brand in supermarkets. Further, regarding the egg-color attribute, consumers are willing to pay more about 32.5 NT$ (≈US$ 1.16) for brown-color eggs if compared to white eggs. This result also corresponds to previous studies [15,21] that consumers prefer to purchase brown eggs over white eggs due to the impression of health concerns and quality issues. However, this result represents the middle consumer group, so it is still not contradictory to the consumers who may prefer the white-color eggs in the small group of class-1.

In addition to the traceability attribute, consumers are willing to pay about 33.4 NT$ (≈US$ 1.19) for fresh egg products with traceability labels in supermarkets. Since consumers in Taiwan are more concerned about food-product originality and safety [88], this study also corresponds to the argument of whether traceability is important in food product labeling. However, the animal welfare attribute presents a higher WTP than any other attribute. This implies that the animal welfare attribute is the most important among these attributes. Consumers in the middle group are willing to pay about 64.2 NT$ (≈US$ 2.29) for fresh eggs with animal welfare attributes in supermarkets. This finding also corresponds to a previous study [13] that consumers do care for animal welfare. Therefore, this study confirms that there are potential markets for animal welfare, traceability, farm brand, and brown-color eggs in supermarkets in Taiwan.

Since the price attribute of the largest consumer group in Table 4 did not show a significant level in the excluding unrealistic choice set model, it may lead to the WTP calculation of the largest consumer group in Table 6 having not shown a significant level in the excluding unrealistic choice set model. This implies that these attributes in the largest consumer group are not identified in the WTP calculations. In other words, consumers in the largest group may focus on other attributes as their preferences. However, regardless of the WTP calculations, consumers in the largest consumer group still care about farm brand, white-color eggs, traceability, and animal welfare attributes.

## 5. Conclusions

Fresh egg products in supermarkets are getting more diversified by promoting different qualities in eggs, such as animal welfare, traceability, farm brand, color of eggs, etc. Since eggs are one of the fundamental elements for daily diet and consumers do care about what they eat, this study attempts to ascertain consumer preferences via the WTP and market segmentations. The CE method was utilized to estimate the market segmentations as well as the most important attributes that may influence consumer preferences. Further, this study also compares the results of including and excluding the unrealistic choice sets in the estimation of the LCM model. According to the values of the goodness of fit, the excluding unrealistic choice set model reveals better goodness of fit than the including unrealistic choice set model. Indeed, the overall outcomes of these two models present differently. Moreover, it is strongly recommended to other studies if the CE method with the choice set situation is adopted; the existing unrealistic choice sets should pay extra attention. With the indication of AIC, BIC, and Log Likelihood, this study only adopts the outcomes of the excluding unrealistic choice set model.

The findings in this research contribute to a better understanding of what motivates Taiwanese consumers to purchase fresh eggs in supermarkets. Among the major attributes, i.e., animal welfare, traceability, farm brand, and brown-color eggs, each attribute reveals a positive preference for certain segments of Taiwanese consumers. Especially, consumers who prefer animal welfare eggs are willing to pay up to about 64.2 NT$ (≈US$ 2.29), which is about twice above from the WTPs of other attributes, i.e., traceability, farm brand, and brown-color eggs. This presents that the animal welfare factor is a hot topic in supermarkets now. The overall results of this study convey strong signals to the egg industry, stakeholders, government, and policymakers about the market potential for brown-color egg, animal welfare, traceability, and farm brand attributes in supermarkets.

Several limitations in this study should be addressed. First, only five attributes are considered in this study, while other potential factors (i.e., nutritional facts, Halal, freshness, etc.) are omitted. Second, this study only focuses on supermarkets in Taiwan, while other major markets, such as traditional markets, are not considered in this study. In order to provide more comprehensive information, a further examination is needed, so government and policymakers would be able to make the relevant policies that encompass the entire consumer demand.

## Figures and Tables

**Table 1:** Attributes and levels in the CE.

Attributes	Levels
Brand	Private Brand
	Farm Brand
Color	Brown
	White
Traceability	Provide Traceability information via QRcode
	No Traceability information provided
Animal welfare	Provide Animal welfare certified label
	No Animal welfare certified label provided
Price	60 NT$/box
	95 NT$/box
	130 NT$/box
	165 NT$/box

Source: Designed by this research.

**Table 2:** The Sample Summary of Variable Description and Statistics (*n* = 1115).

Variables	Descriptions	Mean	S.D.
Age	CV = the respondent’s age	40.20	13.12
Female	DV = 1 if the respondent is female	0.75	0.43
Education	CV = the respondent’s education level in years	15.45	2.17
Retired	DV = 1 if the respondent’s job is retired	0.06	0.23
Housewife	DV = 1 if the respondent’s job is housewife	0.14	0.35
Service	DV = 1 if the respondent’s job is in service sector	0.19	0.39
Manufacture	DV = 1 if the respondent’s job is in manufacture sector	0.10	0.31
Kid at home	DV = 1 if the respondents have kids at home	0.40	0.49
Traditional Markets	CV = Monthly frequency to buy eggs in traditional markets	1.78	2.11
Supermarkets	CV = Monthly frequency to buy eggs in supermarkets	2.25	1.99
Hypermarkets	CV = Monthly frequency to buy eggs in hypermarkets	1.11	1.52

Source: Grouped by this research. Note: DV and CV represent the dummy and continuous variable; S.D. represents the standard deviation.

**Table 3:** Summary of Measures to Determine the Optimal Number of Classes in LCM.

	Number of Classes	Log-Likelihood	AIC	BIC
Including unrealistic choice sets	2	−3013.72	6049.44	6104.62
	3	−2942.95	5919.89	6005.17
	4	−2881.57	5809.14	5924.52
	5	−2861.63	5781.25	5926.73
	6	−2850.11	5770.22	5945.80
	7	−2831.66	5745.31	5950.99
	8	−2833.19	5760.38	5996.16
	9	−2827.88	5761.77	6027.65
Excluding unrealistic choice sets	2	−4234.38	8490.76	8545.94
	3	−4098.08	8230.16	8315.44
	4	−4051.24	8148.48	8263.86
	5	−4017.20	8092.39	8237.88
	6	−3949.99	7969.99	8145.57
	7	−3941.65	7965.31	8170.99
	8	−3939.15	7972.30	8208.09
	9	−3930.94	7967.87	8233.76

Source: Calculated by this research.

**Table 4:** The Results of Parameter Estimates of the LCM.

Variables	Excluding Unrealistic Choice Sets			Including Unrealistic Choice Sets		
	Class 1	Class 2	Class 3	Class 1	Class 2	Class 3
Price	0.002	−0.406 ***	0.000	−0.023 ***	−0.023 ***	0.010 ***
Brand	−2.675 **	13.247 ***	1.299 ***	0.048	1.193 ***	0.867 ***
Color	−2.188 **	13.196 ***	−0.187 *	0.061	1.448 ***	−0.932 ***
Traceability	−1.903 **	13.590 ***	1.134 ***	0.522 *	1.519 ***	0.743 ***
Animal welfare	0.220	26.088 ***	0.910 ***	0.108	2.955 ***	0.160
**Class share**	0.125	0.283	0.591	0.151	0.399	0.450
Age	0.025 ***	−0.012	0	0.025 ***	−0.010	0
Female	−0.431 *	0.366	0	−0.259	0.185	0
Education	0.053	−0.100 **	0	0.030	−0.026	0
Retired	−0.432	0.233	0	−0.436	−0.450	0
Housewife	−0.036	−0.525	0	0.163	−0.021	0
Service	−0.171	−0.212	0	−0.004	0.222	0
Manufacture	−0.375	−0.215	0	−0.457	0.011	0
Kid at home	0.266	−0.226	0	0.068	−0.249	0
Traditional Markets	0.077	0.040	0	0.003	−0.028	0
Supermarkets	−0.080	0.013	0	−0.127 **	0.023	0
Hypermarket	−0.241 ***	0.010	0	−0.234 **	0.067	0
Constant	−2.894 ***	1.065	0	−1.951 *	0.525	0
Observations	13,380			10,035		
Log-Likelihood	−2893.91			−4071.58		
AIC	5865.82			8221.15		
BIC	6147.16			8513.71		

Source: Calculated by this research. ***, **, and * denote statistically significant at the 1%, 5%, and 10%, respectively.

**Table 5:** The summary of socio-demographic factors for the excluding unrealistic model.

Variables	Mean		
	Class 1	Class 2	Class 3
Age *	43.218	38.759	40.376
Female *	0.676	0.778	0.748
Education *	15.542	15.225	15.566
Retired	0.056	0.067	0.050
Housewife	0.169	0.110	0.160
Service	0.176	0.179	0.194
Manufacture	0.092	0.099	0.110
Kid at home	0.465	0.345	0.417
Traditional Markets	1.944	1.957	1.636
Supermarkets	1.859	2.380	2.264
Hypermarket *	0.690	1.198	1.152

Source: Calculated by this research. Note: * indicates the significant variables associated with Table 4.

**Table 6:** The Estimated Outcomes of Average WTP (NT$/10 Eggs).

Variables	Excluding Unrealistic Choice Sets			Including Unrealistic Choice Sets		
	Class 1	Class 2	Class 3	Class 1	Class 2	Class 3
Brand	1125.44	32.59 ***	−3549.67	2.03	51.69 ***	−82.84 ***
Color	920.73	32.46 ***	510.24	2.61	62.74 ***	−89.03 ***
Traceability	800.74	33.43 ***	−3099.16	22.24 **	65.80 ***	−71.01 ***
Animal welfare	−92.73	64.18 ***	−2486.12	4.61	128.00 ***	−15.27

Source: Calculated by this research. Note: *** and ** denote statistically significant at the 1% and 5% significance, respectively.

## Data Availability

The data presented in this research are accessible from the corresponding author upon request. The data are not publicly accessible because research participants did not consent to their data being released publicly.
